# Effects of UV-B Radiation on the Content of Bioactive Components and the Antioxidant Activity of *Prunella vulgaris* L. Spica during Development

**DOI:** 10.3390/molecules23050989

**Published:** 2018-04-24

**Authors:** Yuhang Chen, Xuerong Zhang, Qiaosheng Guo, Li Liu, Chen Li, Liping Cao, Qin Qin, Miao Zhao, Wenming Wang

**Affiliations:** 1 Rice Research Institute, Sichuan Agricultural University, Chengdu 611130, China; chenyuhang221@126.com; 2 Institute of Chinese Medicinal Materials, Nanjing Agricultural University, Nanjing 210095, China; 2015104127@njau.edu.cn (X.Z.); liuli@njau.edu.cn (L.L.); 3 College of Pharmaceutical Sciences, Chengdu Medical College, Chengdu 610500, China; lichen05@sina.com (C.L.); caosiyu818@126.com (L.C.); dolphinbenq@163.com (Q.Q.); redpinkblue@sina.com (M.Z.); 4 Key Laboratory of Small Molecule Special Structure Drugs, Sichuan Institution of Higher Education/Chengdu Medical College, Chengdu 610500, China

**Keywords:** *Prunella vulgaris* L., UV-B, bioactive components, antioxidant activity

## Abstract

The effects of UV-B radiation on the content of bioactive components and the antioxidant activity of *Prunella vulgaris* L. spica during development were studied. The experimental design involved two levels of UV-B radiation intensity (0 and 120 μW cm^−2^ nm^−1^). The results showed that the contents of total flavonoids, rosmarinic acid, caffeic acid and hyperoside, as well as the antioxidant capacities (DPPH^●^ and ABTS•+ scavenging activities), in the spicas significantly decreased during spica development. The content of salviaflaside in the spicas significantly increased during development. The highest contents of total flavonoids, rosmarinic acid, and caffeic acid and the highest antioxidant activities were found in spicas in the full-flowering stage, while the highest content of hyperoside was found in spicas in the bud stage. In addition, the highest content of salviaflaside was found in spicas in the mature-fruiting stage. UV-B radiation significantly promoted the synthesis of secondary metabolites, increased the contents of the main bioactive components in the three developmental stages of isolated dried spicas, and significantly increased the DPPH^●^ and ABTS•+ scavenging activities of *P. vulgaris* spicas in the mature-fruiting stage. Moreover, the total flavonoids content was positively correlated with the DPPH^●^ and ABTS•+ scavenging activities, and the correlation with the DPPH^●^ scavenging activity was very strong. This result shows that the highest contents of the main bioactive components in the spicas were not all found in the same developmental stages of *P. vulgaris*. Our research revealed that the best stage for harvesting *P. vulgaris* spica was between the bud stage and the full-flowering stage since harvesting at this point provides a higher content of bioactive components and a higher antioxidant capacity, which is relevant for medicinal applications.

## 1. Introduction

The depletion of the stratospheric ozone layer has become a serious global environmental problem that has led to an increase in the amount of ultraviolet-B radiation (UV-B, 280–320 nm) reaching the Earth’s surface, and the amount of UV-B radiation on the Earth’s surface will continue to increase in the coming decades [1,2]. In general, many studies have reported that UV-B radiation has harmful effects on the growth, physiology and productivity of plants [3,4,5]. However, in recent years, some studies have found that UV-B radiation can have beneficial effects on the secondary metabolism of medicinal plants [4,5]. Most medicinally bioactive components in medicinal plants are secondary metabolites. Thus, it is necessary to determine whether increasing exposure to UV-B radiation can be used as a simple and environmentally friendly method for increasing the contents of bioactive components in medicinal plants.

Previous studies have shown that UV-B radiation can induce secondary metabolism processes while increasing the contents of bioactive components in medicinal plants [6,7]. However, these experiments evaluated UV-B radiation during each plant’s growth period, which was operationally difficult (especially in places without a stable supply of electricity) and required a large economic investment in production. Hence, further studies to explore the optimal method of UV-B radiation for improving the content of bioactive compounds in medicinal plants are necessary.

A few studies have reported the effects of UV-B radiation on isolated organs of medicinal plants to achieve a more efficient and deeper application of UV-B radiation to the medicinal plants [8,9]. A previous study showed that UV-B radiation dramatically accelerated the accumulation of seven compounds in freshly collected flower buds of *Lonicera japonica* Thunb [10]. A similar study indicated that UV-B radiation promoted the accumulation of secondary metabolites in postharvest fresh leaves of *Ginkgo biloba* L. [11]. Another study reported that UV-B radiation significantly increased the contents of bioactive components in fresh medicinal chrysanthemum flowers isolated by various methods [12]. To the best of our knowledge, few experimental studies have explored the effects of UV-B radiation on the contents of bioactive components and the antioxidant activity of the ethanol extract during spica development of *Prunella vulgaris* L. Additionally, researchers have only recently started paying attention to the effects of UV-B radiation on freshly isolated organs of medicinal plants, but few studies have been conducted on the bioactive components of dried isolated organs. Therefore, further studies should be done in order to better evaluate the effect of the application of UV-B radiation on dried isolated organs of medicinal plants to facilitate the harvesting, processing, storage and transportation of Chinese medicinal materials.

*P. vulgaris* is an important medicinal plant commonly found in Europe, South Asia and Northeast Asia [5,13]. The dried spicas of *P. vulgaris*, Prunellae Spica, are occasionally used for the treatment of pulmonary tuberculosis, infectious hepatitis, hypertension, and mastitis [14]. The main bioactive components of Spica Prunellae are phenolic acids (e.g., rosmarinic acid, caffeic acid and salviaflaside) and flavonoids [15,16]. This study focuses on the effects of UV-B radiation on the contents of bioactive components and the antioxidant activity of the ethanol extract during the three developmental stages of *P. vulgaris* to determine which stage is most sensitive to UV-B radiation and identify the optimum stage for harvesting based on the contents of bioactive components and the antioxidant activity of the ethanol extract of the spicas. This will elucidate the effects of UV-B radiation on postharvest dried spica of *P. vulgaris*.

## 2. Results

### 2.1. Effects of UV-B Radiation on the Contents of Total Flavonoids, Rosmarinic Acid and Caffeic Acid

**Figure 1:**
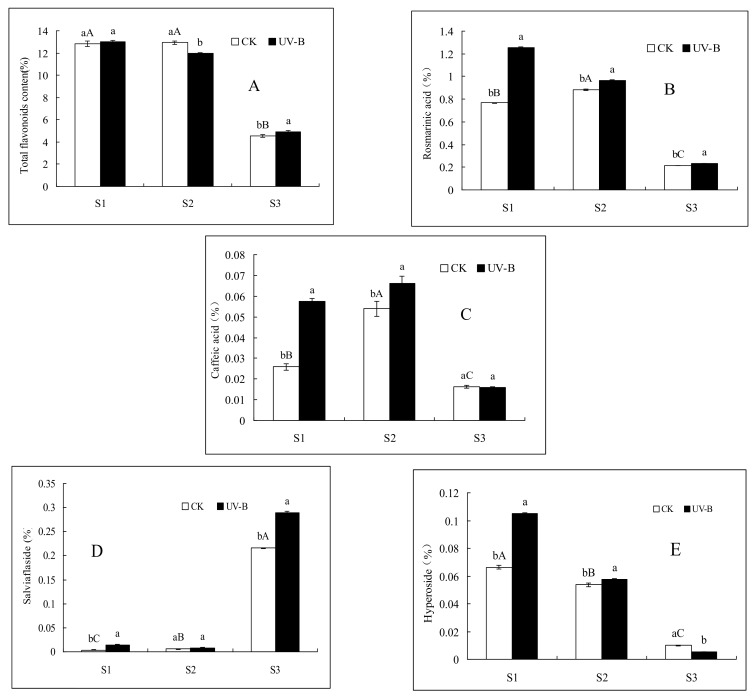
Effects of UV-B radiation on the contents of total flavonoids (**A**); rosmarinic acid (**B**); caffeic acid (**C**); salviaflaside (**D**) and hyperoside (**E**) in *P. vulgaris* spicas in three developmental stages. Control group (CK): 0 μW cm^−2^ nm^−1^; UV-B: 120 μW cm^−2^ nm^−1^; S1, the bud stage; S2 the full-flowering stage; and S3, the mature-fruiting stage. Different lower-case letters indicate significant differences between CK treatments and UV-B treatments, and different capital letters indicate significant differences between the three developmental stages of *P. vulgaris* (*p* < 0.05).

The contents of total flavonoids, rosmarinic acid and caffeic acid were significantly different in the spicas from the three developmental stages, and spicas in the full-flowering stage had the highest contents (Figure 1). The contents of total flavonoids, rosmarinic acid and caffeic acid in the spicas were found to decrease in the order full-flowering stage > the bud stage > the mature-fruiting stage. The contents of rosmarinic acid and caffeic acid in the spica in the three developmental stages were significantly increased by exposure to UV-B radiation (the content of caffeic acid was not significantly different in the mature-fruiting stage). In addition, an increased content of total flavonoids in spica in the bud stage of development, as well as in the mature-fruiting stage, was observed, but significantly decreased levels were observed for samples in the full-flowering stage.

### 2.2. Effects of UV-B Radiation on the Contents of Salviaflaside and Hyperoside

The contents of salviaflaside and hyperoside were significantly different in the spica in the three stages of development (Figure 1). The salviaflaside content significantly increased during spica development, while the hyperoside content significantly decreased during spica development. The salviaflaside content decreased in the order the mature-fruiting stage > the full-flowering stage > the bud stage, and the hyperoside content decreased in the order the bud stage > the full-flowering stage > the mature-fruiting stage. UV-B radiation significantly promoted the accumulation of salviaflaside and hyperoside in the three stages of spica development (except for salviaflaside in the full-flowering stage and hyperoside in the mature-fruiting stage).

### 2.3. Effects of UV-B Radiation on the Antioxidant Activities

Both the DPPH^●^ and ABTS•+ radical scavenging activities significantly decreased during spica development with the highest values being recorded in the full-flowering stage and lowest in the mature-fruiting stage (Table 1). UV-B radiation significantly decreased the DPPH^●^ scavenging activity in the full-flowering stage and slightly decreased the activities in the bud stage and full-flowering stage. Meanwhile, UV-B radiation significantly enhanced the ABTS•+ radical scavenging activity in the mature-fruiting stage and significantly decreased the activity in the bud stage. No significant difference was observed for the full-flowering stage.

### 2.4. Correlation Analysis

Significant positive correlations were observed between the total flavonoids content and the DPPH^●^ scavenging activity (R = 0.999*/R = 0.999*) as well as between the total flavonoids content and the ABTS•+ scavenging activity (R = 0.999*/R = 0.720) in dried spicas of *P. vulgaris* (Table 2). For the control treatment, strong correlation between the DPPH^●^ scavenging activities and the contents of rosmarinic acid (R = 0.993*), caffeic acid (R = 0.727) and hyperoside (R = 0.967) were detected. However, for UV-B treatments, the contents of rosmarinic acid, caffeic acid, hyperoside and salviaflaside were not correlated with values of the DPPH^●^ and ABTS•+ free radical scavenging activities (R values ranged from 0.187 to 0.312 and from 0.132 to 0.224, respectively). Additionally, for the control treatment, a significant negative correlation between the salviaflaside content and the DPPH^●^ scavenging activity (R = −0.999*) was observed.

## 3. Discussion

Our previous study found that UV-B radiation could affect the morphology, physiological traits and accumulation of bioactive compounds in spicas of *P. vulgaris* [5]. The present research showed that there were significant differences in the contents of bioactive compounds and the antioxidant activities of *P. vulgaris* spicas during the three developmental stages, and the main bioactive compounds in the isolated dried spicas in the different developmental stages were significantly affected by the UV-B radiation.

Some related studies have indicated that the contents of secondary metabolites in medicinal plants can affect their pharmacological behaviour [17]. Phenolic acids and flavonoids the are major bioactive compounds in spicas of *P. vulgaris* [18]. The present study proved that the contents of total flavonoids, rosmarinic acid and caffeic acid significantly differed in the spicas in the three stages of development, and the highest contents were found in the full-flowering stage. Previous studies revealed that the contents of total flavonoids and rosmarinic acid in *P. vulgaris* spicas were highest in the full-flower stage [19,20]. In addition, a similar study reported that the caffeic acid content was higher in the earlier developmental stages of spicas in *P. vulgaris* than in the latter stages [21]. The data further showed that the contents of rosmarinic acid and caffeic acid were higher in spicas in the bud and full-flowering stages. Except for caffeic acid in the mature-fruiting stage and the total flavonoids in the bud and full flowering stages, UV-B radiation significantly promoted the accumulation of flavonoids, rosmarinic acid and caffeic acid in the *P. vulgaris* spicas in the three developmental stages.

Hyperoside and salviaflaside are also important bioactive compounds in *P. vulgaris*. Hyperoside, a compound that is ubiquitous in plant tissues, protects plants from oxidative damage by removing active oxygen as a substrate for enzymes [22]. Meanwhile, salviaflaside is a major phenolic glycoside component in spicas *P. vulgaris* that is formed by the intermolecular condensation of two phenylpropanoid derivatives [14]. In this study, the content of hyperoside significantly decreased during spica development, and its content was highest in the bud stage. However, the content of salviaflaside significantly increased during spica development, and its content was highest in the mature-fruiting stage. A previous study reported that the content of hyperoside in *Hibiseu manihot* L. was highest in the bud stage [23]. In addition, a similar study reported that the hyperoside content in the early developmental stages of flowers in *Hypericum triquetrifolium* Turra was significantly higher than those in the vegetative and fruiting stages [24]. In this study, the content of hyperoside in the spica in the early stages of development was higher than in that in the latter stages, suggesting that secondary metabolism was more active during the early stages of spica development. UV-B radiation significantly increased the contents of hyperoside and salviaflaside in the spicas in the three stages of development (except for hyperoside in the mature-fruiting stage and salviaflaside in the full-flowering stage), which facilitated the accumulation of secondary metabolites in spicas.

Free radicals can be generated by oxidation reactions. Antioxidants are molecules that primarily alleviate or prevent oxidation chain reactions from terminating in vitro and in vivo [25]. In biological systems, the pathogenesis of many diseases is related to oxidative stress [26,27]. Phenolic acids and flavonoids are well-known to have beneficial health effects, such as antioxidative, antimicrobial, anti-inflammatory, and antimutagenic properties, for various organisms [14]. In this study, we measured the antioxidant capacities of two ethanol extracts by the DPPH^●^ and ABTS•+ assays [28,29]. The antioxidant activity of *P. vulgaris* spicas showed a similar fluctuation with growth stage as was seen in the phenolic and flavonoid contents since the DPPH^●^ and ABTS•+ scavenging activities were positively correlated with the contents of bioactive compounds in the spica except in case of the salviaflaside (Table 2). Indeed, the highest antioxidant activity in *P. vulgaris* spicas was found in the full-flowering stage, which is consistent with the results reported in the previous study [30]. Previous studies found a positive relationship between the contents of total phenolics and total flavonoids with the DPPH^●^ and ABTS•+ scavenging activities of other plants [31,32]. In addition, we found a strong positive correlation between the total flavonoids content and the DPPH^●^ scavenging activity (R = 0.999*/R = 0.999*) as well as between the total flavonoids content and the ABTS•+ scavenging activity (R = 0.999*/R = 0.720) (Table 2). In addition, a previous study reported that the DPPH^●^ scavenging activity did not increase with increasing total phenolics content in *P. vulgaris* spicas [33]. For the control treatment, we found a strong correlation between the DPPH^●^ scavenging activity and the rosmarinic acid content (R = 0.993*), the caffeic acid content (R = 0.727) and the hyperoside content (R = 0.967). The difference may be because in addition to the effect from the total phenolics, the main bioactive components (e.g., rosmarinic acid, caffeic acid and hyperoside) could also contribute to the antioxidant capacity of *P. vulgaris*. For the UV-B treatments, the bioactive components (e.g., rosmarinic acid, caffeic acid, hyperoside and salviaflaside) were not correlated with the DPPH^●^ and ABTS•+ free radical scavenging activities (R values ranged from 0.187 to 0.312 and from 0.132 to 0.224, respectively). Previous studies have reported that the antioxidant capacity of citrus flesh as determined by the DPPH^●^ method was strongly correlated with the total flavonoid contents, suggesting that flavonoids acted as free radical scavengers in *P. vulgaris* spicas*.* Similar results were presented in an earlier work [34].

## 4. Materials and Methods

### 4.1. Plant Material and Growth Conditions

**Figure 2:**
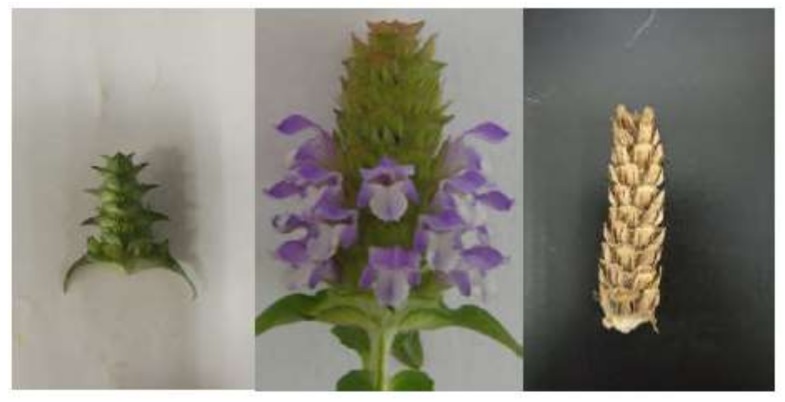
Spica development periods of *P. vulgaris* (from left to right: stage 1: bud stage, tage 2: full-flowering stage, and stage 3: mature-fruiting stage).

This study was carried out at the Chengdu Medical College, Chengdu, PR. China. *P. vulgaris* seeds were collected in June 2016 from the experimental planting station at Chengdu Medical College, Sichuan Province, and then sown in the experimental farmland in October 2016. Routine field management conditions were used during the whole growth period of *P. vulgaris*. Fresh spicas were divided into three groups according to their development stage (Figure 2): bud stage, 3–4 layers of green calyx formed, and the crown not yet opened (bud length 1–2 cm); full-flowering stage, 5–10 layers of calyx formed, and the crown being opened 60–70% (inflorescence length 3–5 cm); mature-fruiting stage, the brown calyx formed, and the crown being faded (inflorescence length 6–7 cm).

### 4.2. UV-B Treatments

The fresh spicas were harvested, dried for 12 h at 70 °C, and then treated with UV-B radiation for 120 min. The UV-B irradiance was measured with a portable light meter (UV-340A, Lutron, Taiwan). This study used two levels of UV-B radiation intensity: (1) Control group (CK): 0 μW cm^−2^ nm^−1^, and (2) UV-B intensity: 120 μW cm^−2^ nm^−1^. After UV-B radiation, the dried spicas were pulverized and sieved through a 60-mesh sieve prior to analyses. Each treatment was carried out in triplicate.

UV-B radiation was applied using UV-B fluorescent lamps (40 W, 305 nm, Beijing Electric Light Source Research Institute, Beijing, China) mounted in metal frames. In the laboratory, UV-C radiation from the lamps was excluded by wrapping the tubes with 0.125-mm thick cellulose diacetate film (Lucky Films Co. Ltd., Baoding, China), which filters out the UV-C (<280 nm) irradiation from the UV-B treatment.

### 4.3. Determination of Total Flavonoids Content

The powder of the dried spica (1.0 g) was extracted with 35% ethanol (10 mL) at 86 °C in a water bath and subjected to circumfluence extraction for 3.5 h. Then, 1 mL of the above extract was mixed with 50 mg mL^−1^ NaNO_3_ (0.7 mL) in a test tube for 7 min. After that, 100 mg mL^−1^ AlCl_3_ (0.3 mL) was added, and the solution was mixed for 6 min. Finally, 5.0 mL of 1 mol L^−1^ NaOH solution was added, and the absorbance was measured at 510 nm using a 752 UV-visible spectrophotometer (Shanghai Jinghua Technological Instrument, Shanghai, China). Rutin was used as a standard to prepare a calibration curve, and the results are expressed as mg rutin equivalents per 100 mg dry weight of spicas (%).

### 4.4. Determination of the Caffeic Acid, Salviaflaside, Rosmarinic Acid and Hyperoside Contents

**Figure 3:**
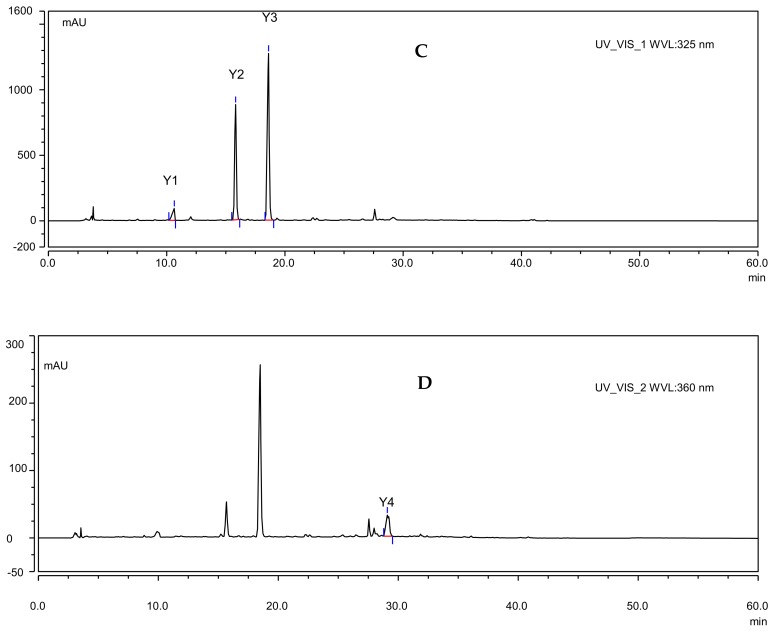

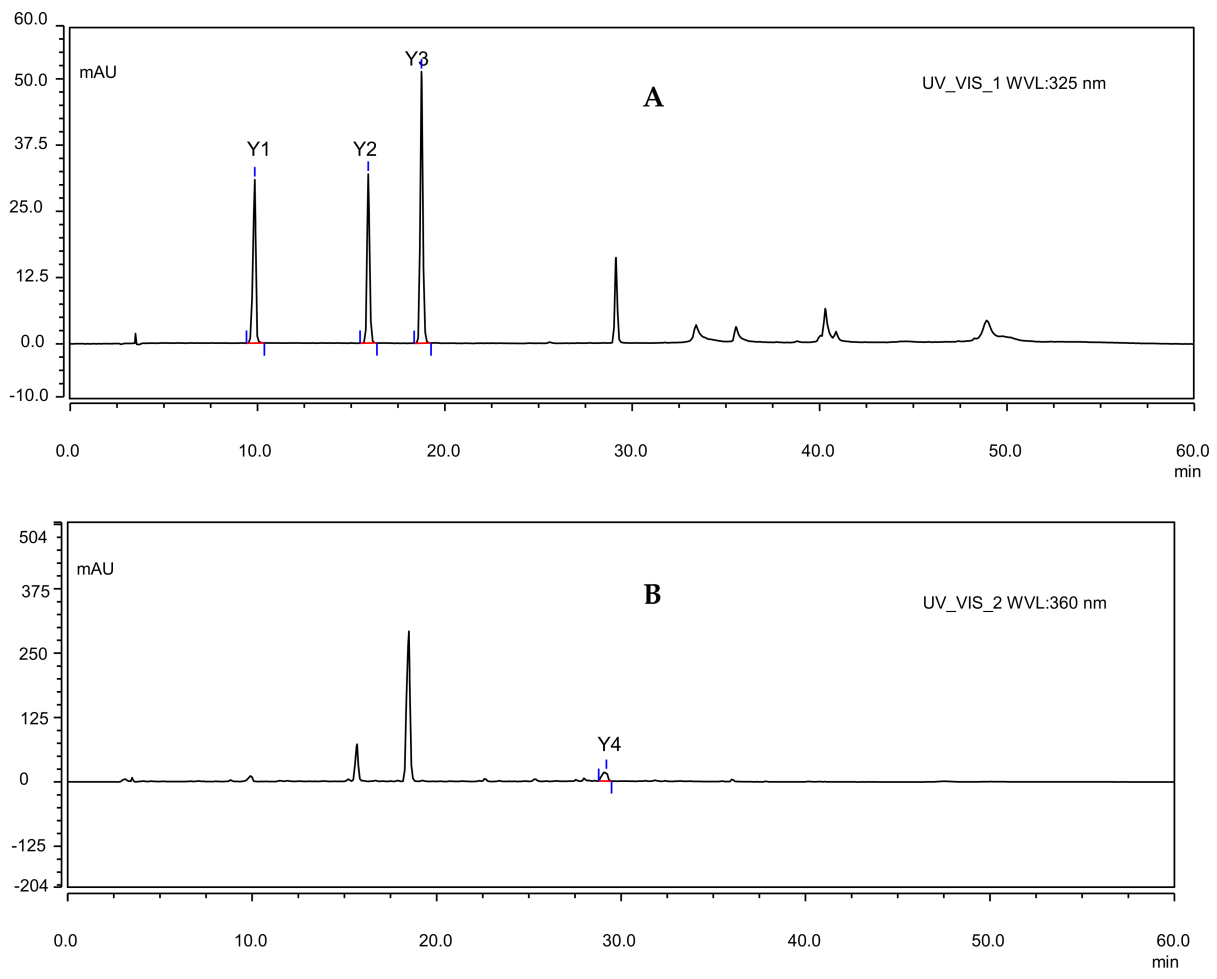
HPLC chromatograms of authentic standards and the methanol extract of *P. vulgaris* spicas. (**A**,**B**) UV chromatograms of the 4 authentic standards at 325 nm and 360 nm. (**C**,**D**) UV chromatograms of methanol extract at 325 nm and 360 nm. (1) caffeic acid (Y1); (2) salviaflaside (Y2); (3) rosmarinic acid (Y3); and (4) hyperoside (Y4).

The powder of dried spica (2.0 g) was mixed with 80% methanol (15 mL) in an ultrasonic bath for 35 min at room temperature, and then the extracted solution was centrifuged at 12,000 rpm for 15 min. The supernatant was passed through a 0.45-μm organic membrane filter before HPLC analysis. The extract (10 μL) was analysed on a Dionex UltiMate 3000 HPLC System (Dionex Corp., Sunnyvale, CA, USA) equipped with a Uranus C18 (250 mm × 4.6 mm) and a diode array detector (DAD-3000). The mobile phase consisted of methanol (solvent A) and 0.2% NaH_2_PO_4_ solution (solvent B), the flow rate was 0.8 mL min^−1^ and the following multilinear gradient was used: 20–40% A (0–20 min), 40–70% A (20–35 min), 70–90% A (35–45 min), and 90–20% A (45–60 min). The detection wavelengths were 325 nm and 360 nm (325 nm detection wavelength for caffeic acid, salviaflaside and rosmarinic acid and 360 nm detection wavelength for hyperoside). The column oven temperature was 30 °C, and the total run time was 60 min. Each peak was identified based on its retention time and comparison to chromatographs of authentic standards. The contents of the bioactive compounds were calculated according to calibration curves of the standards (Table 3), and the results are expressed as mg per 100 mg dry weight of spicas (%). HPLC chromatograms of the mixed standard stock solution and an extract of *P. vulgaris* are shown in Figure 3.

### 4.5. DPPH Free Radical Scavenging Assay

The antioxidant scavenging activity was studied using the DPPH^●^ spectrophotometric method described in a previous study with some modifications [35]. The dried powder of spica (1.0 g) was extracted with 70% ethanol (30 mL) using ultrasound extraction for 30 min at 70 °C. An aliquot of the sample solution (1.0 mL) was added to 4 mL of a methanolic solution of DPPH^●^ (0.004%, *w*/*v*, 50 μg mL^−1^). Meanwhile, a 1.0 mL aliquot of the sample solution was added to 4 mL of methanol as the blank, and a 1.0 mL aliquot of methanol was added to 4 mL of methanolic DPPH^●^ as the control. After incubation at 25 °C for 30 min, the absorbance was measured at 517 nm using a 752 UV-visible spectrophotometer (Shanghai Jinghua Technological Instrument, Shanghai, China). All measurements were performed in triplicate and were taken while protected from light. The DPPH^●^ scavenging effect was calculated as follows:

DPPH^●^ scavenging effect (%) = [Acontrol − (Asample − Ablank)/Acontrol] × 100%

where Acontrol is the absorbance of 4 mL of DPPH^●^ radical + 1 mL of methanol, Asample is the absorbance of 4 mL of DPPH• radical + 1 mL of sample, and Ablank is the absorbance of 4 mL of methanol + 1 mL of sample.

### 4.6. Trolox Equivalent Antioxidant Capacity Assay (TEAC Assay)

The radical scavenging capacity for the ABTS•+ radical cation was determined following the procedure previously reported with some modifications [35]. The radical was generated by reacting a 7 mmol L^−1^ solution of ABTS•+ with 2.45 mmol L^−1^ potassium persulfate. The final working solution of ABTS•+ was obtained after allowing this mixture to react for 12 to 16 h at ambient temperature (23 °C) in darkness. The ABTS•+ reagent was diluted with phosphate buffered saline (pH 7.4) to reach an absorbance of 0.700 ± 0.005 at 734 nm. For the spectrophotometric measurements of the samples, 3.9 mL of dilute ABTS•+ solution and 0.1 mL of sample were mixed. A calibration curve was prepared using Trolox at concentrations ranging from 80 to 1600 μmol L^−1^ in methanol. Dilute ABTS•+ solution (3.9 mL) mixed with 0.1 mL of water was used as the control. The absorbance was measured at 734 nm after 6 min using a 752 UV-visible spectrophotometer (Shanghai Jinghua Technological Instrument, Shanghai, China). The total antioxidant capacities of the samples are expressed as equivalents of Trolox (TEAC) per 1 g of dry matter of the sample (μmol g^−1^). All measurements were performed in triplicate, and the ABTS•+ scavenging effect was calculated as follows:

ABTS•+ scavenging effect (%) = [(1 − Asample)/Acontrol] × 100%

where Asample is the absorbance of 3.9 mL of diluted ABTS•+ + 0.1 mL of sample and A control is the absorbance of 3.9 mL of diluted ABTS•+ + 0.1 mL of water.

Trolox was used to prepare a standard calibration curve, and the results are expressed as TEAC. The concentration of antioxidants giving the same percentage inhibition of ABTS•+ as that achieved with 1 mM Trolox was regarded as the TEAC.

### 4.7. Statistical Analyses

Each test was conducted in triplicate. All data are expressed as the means ± SD, and one-way analysis of variance (ANOVA) followed by Duncan’s multiple range tests (*p* < 0.05) were conducted with SPSS 17.0 software (SPSS, Chicago, IL, USA). SPSS 17.0 software was also used to determine the correlation coefficients.

## 5. Conclusions

The contents of bioactive components and in vitro antioxidant activities in dried spica were dependent on the developmental phases of *P. vulgaris*. UV-B radiation significantly promoted the synthesis of secondary metabolites and increased the contents of the main bioactive components in the isolated dried spicas from the three developmental stages and significantly increased the DPPH^●^ and ABTS•+ scavenging activities of *P. vulgaris* spicas in the mature-fruiting stage. Moreover, the total flavonoids content was positively correlated with the DPPH^●^ and ABTS•+ scavenging activities, and the correlation with the DPPH^●^ scavenging activity was very strong. These results showed that the highest contents of the main bioactive components in the spicas were not all found in the same developmental stages of *P. vulgaris*. Our research revealed that the best stage for harvesting *P. vulgaris* spica was between the bud stage and the full-flowering stage as this stage provides higher contents of bioactive components and higher antioxidant capacities, which are relevant for medicinal applications.

## Figures and Tables

**Table 1:** The DPPH^●^ and ABTS•+ radical scavenging activities in the ethanol extracts of *P. vulgaris* spicas at the three developmental stages under the control and UV-B treatments.

Antioxidant Index	UV-B Dosage (μW cm^−2^ nm^−1^)	Bud Stage	Full-Flowering	Mature-Fruiting
DPPH^●^ (%)	0	90.41 ± 0.23 aA	91.24 ± 0.10 aA	74.36 ± 2.90 aB
	120	90.35 ± 0.83 aA	88.61 ± 0.34 bA	70.49 ± 2.62 aB
TEAC (mmol L^−1^ Trolox)	0	1.31 ± 0.01 aA	1.35 ± 0.02 aA	0.80 ± 0.05 bB
	120	1.13 ± 0.05 bB	1.33 ± 0.05 aA	1.00 ± 0.02 aC

Each value is presented as the mean ± SD (n = 3). Different lower-case letters indicate significant differences between the CK and UV-B treatments, and different capital letters indicate significant differences between the three developmental stages of P. vulgaris (*p* < 0.05).

**Table 2:** Correlation analysis between the contents of the bioactive compounds and the DPPH^●^ and ABTS•+ radical scavenging activities of the ethanol extracts of *P. vulgaris* spicas at three developmental stages of under the control and UV-B treatments.

Antioxidant Index	UV-B Dose (μW cm^−2^ nm^−1^)	Rosmarinic Acid (%)	Caffeic Acid (%)	Hyperoside (%)	Salviaflaside (%)	Total Flavonoids (%)
DPPH^●^ (%)	0	0.993 *	0.727	0.967	−0.999 *	0.999 *
	120	0.187	0.189	0.229	0.312	0.999 *
TEAC (mmol L^−1^ Trolox)	0	0.191	0.202	0.244	0.334	0.999 *
	120	0.132	0.136	0.163	0.224	0.720

* The level of significance is indicated as follows: 0.01 < *p* < 0.05.

**Table 3:** Calibration curves of 5 standard chemicals.

Standard Chemicals	Regression Equation	R^2^	Linear Range
Total flavonoids	*y* = 0.5899*x* + 0.0199	0.9999	0.00–2.00
Rosmarinic acid	*y* = 39999*x* + 0.0031	0.9999	0.000209–0.001045
Caffeic acid	*y* = 49130*x* − 0.0422	0.9999	0.000108–0.00054
Salviaflaside	*y* = 23282*x* − 0.044	0.9999	0.00022–0.0011
Hyperoside	*y* = 33699*x* − 0.0094	0.9999	0.00011–0.00055

y: peak area; x: concentration of chemicals (mg mL^−1^).

## Supplementary Materials

Supplementary File 1
