# Supplementary material for: Effects of UV-B Radiation on the Content of Bioactive Components and the Antioxidant Activity of Prunella vulgaris L. Spica during Development
Source: Molecules. 2018 Apr 24;23(5):989. doi: 10.3390/molecules23050989 (PMC6099561; doi:10.3390/molecules23050989)
Supplement: Supplementary File 1 [file molecules-23-00989-s001.zip › figure and table PDF╬─╝■ ╡┌╢■┤╬╨▐╕─╕σ/Figure 1 Revised version.doc.pdf]

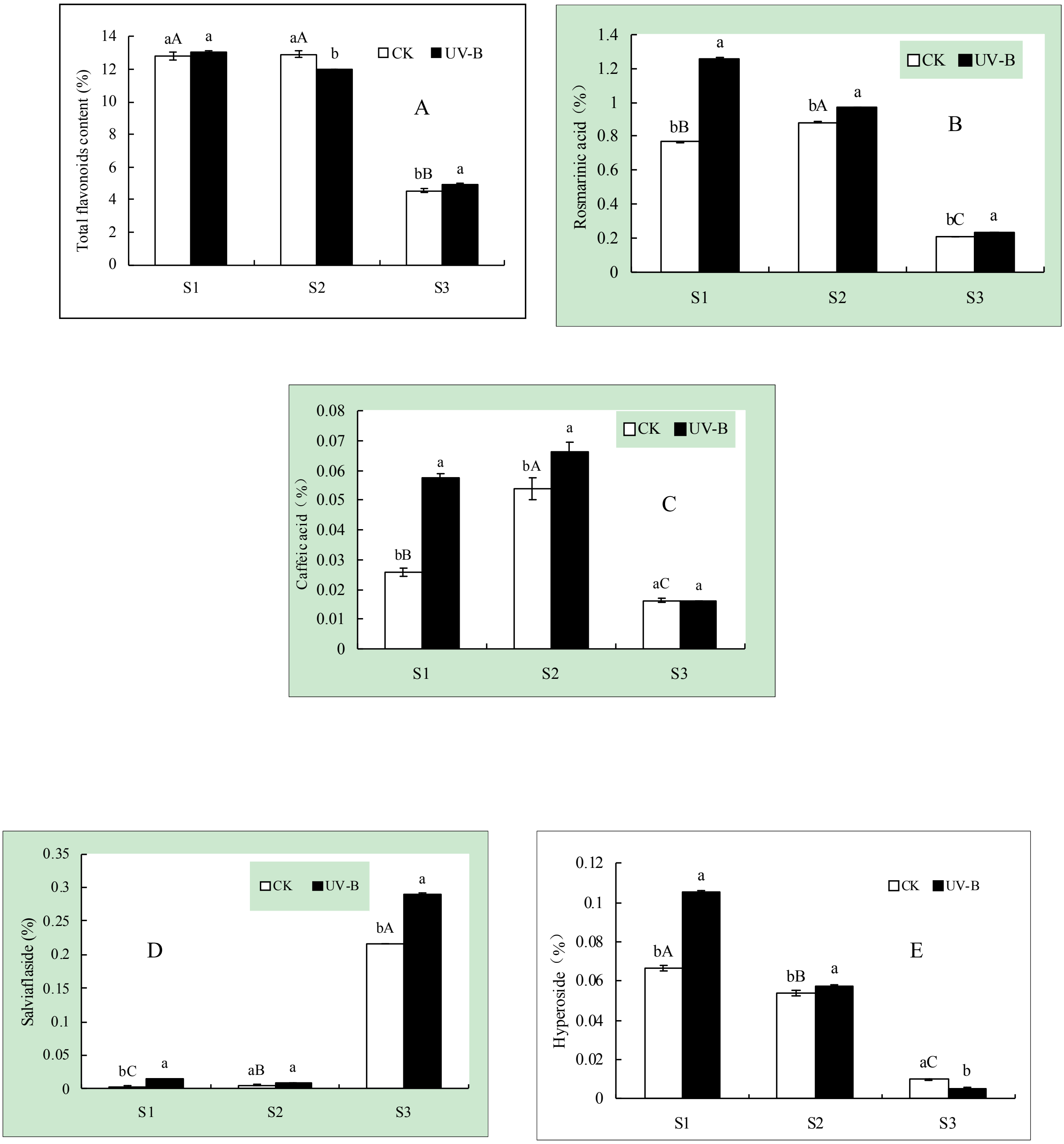

Figure 1. Effects of UV-B radiation on the contents of total flavonoids (A), rosmarinic acid (B), caffeic acid (C), salviaflaside (D) and hyperoside (E) in *P. vulgaris* spicas in three

developmental stages. CK: 0 μW cm^{-2} nm^{-1}; UV-B: 120 μW cm^{-2} nm^{-1}; S1, the bud stage; S2 the full-flowering stage; and S3, the mature-fruiting stage. Different lower-case letters indicate significant differences between CK treatments and UV-B treatments, and different capital letters indicate significant differences between the three developmental stages of *P. vulgaris* ($p < 0.05$).
