# Supplementary material for: Effects of UV-B Radiation on the Content of Bioactive Components and the Antioxidant Activity of Prunella vulgaris L. Spica during Development
Source: Molecules. 2018 Apr 24;23(5):989. doi: 10.3390/molecules23050989 (PMC6099561; doi:10.3390/molecules23050989)
Supplement: Supplementary File 1 [file molecules-23-00989-s001.zip › figure and table PDF╬─╝■ ╡┌╢■┤╬╨▐╕─╕σ/Figure 2.doc.pdf]

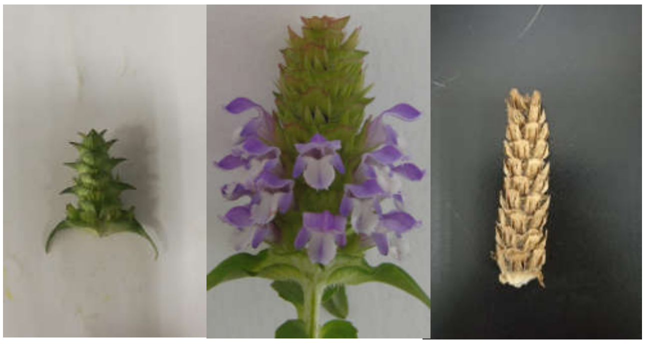

Figure 2. Spica development periods of *P. vulgaris* (from left to right: stage 1: bud stage, stage 2: full-flowering stage, and stage 3: mature-fruiting stage).
