# Supplementary material for: Effects of UV-B Radiation on the Content of Bioactive Components and the Antioxidant Activity of Prunella vulgaris L. Spica during Development
Source: Molecules. 2018 Apr 24;23(5):989. doi: 10.3390/molecules23050989 (PMC6099561; doi:10.3390/molecules23050989)
Supplement: Supplementary File 1 [file molecules-23-00989-s001.zip › figure and table PDF╬─╝■ ╡┌╢■┤╬╨▐╕─╕σ/Figure 3.doc.pdf]

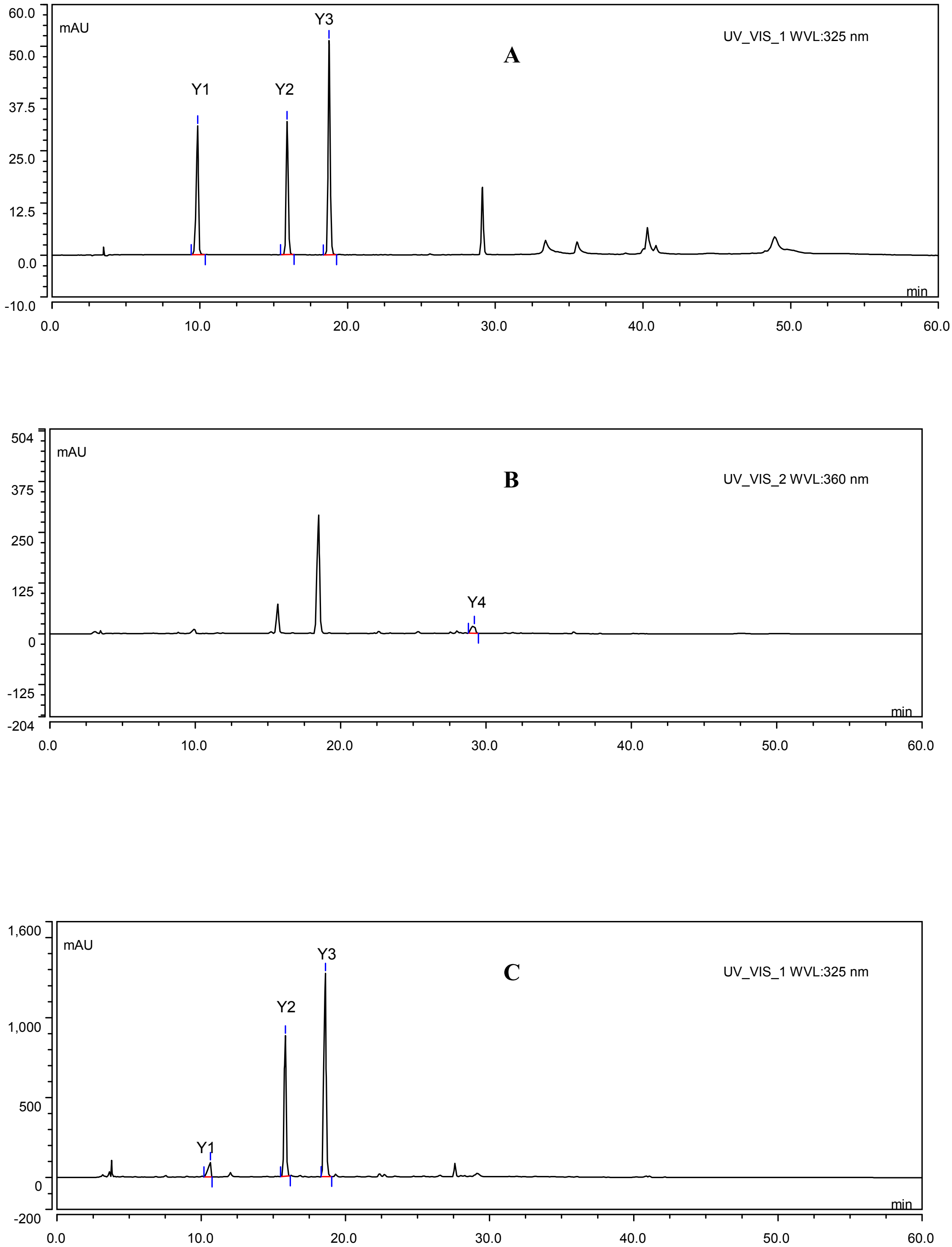

A
mAU
UV_VIS_1 WVL:325 nm
Y1
Y2
Y3
60.0
50.0
37.5
25.0
12.5
0.0
-10.0
min
0.0
10.0
20.0
30.0
40.0
50.0
60.0
B
mAU
UV_VIS_2 WVL:360 nm
Y4
504
375
250
125
0
-125
-204
min
C
mAU
UV_VIS_1 WVL:325 nm
Y1
Y2
Y3
1,600
1,000
500
0
-200
min

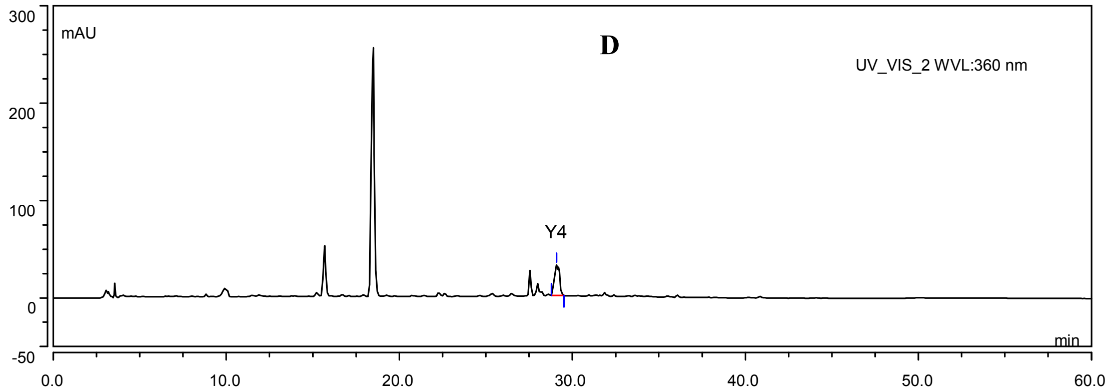

Figure 3. HPLC chromatograms of authentic standards and the methanol extract of *P. vulgaris* spicas. (**A-B**) UV chromatograms of the 4 authentic standards at 325 nm and 360 nm. (**C-D**) UV chromatograms of methanol extract at 325 nm and 360 nm. (1) caffeic acid (Y1); (2) salviaflaside (Y2); (3) rosmarinic acid (Y3); and (4) hyperoside (Y4).
