# Supplementary material for: Effects of UV-B Radiation on the Content of Bioactive Components and the Antioxidant Activity of Prunella vulgaris L. Spica during Development
Source: Molecules. 2018 Apr 24;23(5):989. doi: 10.3390/molecules23050989 (PMC6099561; doi:10.3390/molecules23050989)
Supplement: Supplementary File 1 [file molecules-23-00989-s001.zip › figure and table PDF╬─╝■ ╡┌╢■┤╬╨▐╕─╕σ/Table 1.doc.pdf]

Table 1. The DPPH$^{\bullet}$ and ABTS•+ radical scavenging activities in the ethanol extracts of *P. vulgaris* spicas at the three developmental stages under the control and UV-B treatments.

Antioxidant index	UV-B dosage ($\mu W\ cm^{-2}\ nm^{-1}$)	Bud stage	Full-flowering	Mature-fruiting
DPPH$^{\bullet}$ (%)	0	90.41 ± 0.23 aA	91.24 ± 0.10 aA	74.36 ± 2.90 aB
	120	90.35 ± 0.83 aA	88.61 ± 0.34 bA	70.49 ± 2.62 aB
TEAC (mmol L^{-1} Trolox)	0	1.31 ± 0.01 aA	1.35 ± 0.02 aA	0.80 ± 0.05 bB
	120	1.13 ± 0.05 bB	1.33 ± 0.05 aA	1.00 ± 0.02 aC

Each value is presented as the mean ± SD (n = 3). Different lower-case letters indicate significant differences between the CK and UV-B treatments, and different capital letters indicate significant differences between the three developmental stages of *P. vulgaris* ($p < 0.05$).
