# Supplementary material for: Effects of UV-B Radiation on the Content of Bioactive Components and the Antioxidant Activity of Prunella vulgaris L. Spica during Development
Source: Molecules. 2018 Apr 24;23(5):989. doi: 10.3390/molecules23050989 (PMC6099561; doi:10.3390/molecules23050989)
Supplement: Supplementary File 1 [file molecules-23-00989-s001.zip › figure and table PDF╬─╝■ ╡┌╢■┤╬╨▐╕─╕σ/Table 2.doc.pdf]

Table 2. Correlation analysis between the contents of the bioactive compounds and the DPPH• and ABTS•+ radical scavenging activities of the ethanol extracts of *P. vulgaris* spicas at three developmental stages of under the control and UV-B treatments

Antioxidant index	UV-B dose ($\mu W\ cm^{-2}\ nm^{-1}$)	Rosmarinic acid (%)	Caffeic acid (%)	Hyperoside (%)	Salviaflaside (%)	Total flavonoids (%)
DPPH• (%)	0	0.993*	0.727	0.967	-0.999*	0.999*
	120	0.187	0.189	0.229	0.312	0.999*
TEAC (mmol L^{-1} Trolox)	0	0.191	0.202	0.244	0.334	0.999*
	120	0.132	0.136	0.163	0.224	0.720

* The level of significance is indicated as follows: $0.01 < p < 0.05$.
