# Supplementary material for: Effects of UV-B Radiation on the Content of Bioactive Components and the Antioxidant Activity of Prunella vulgaris L. Spica during Development
Source: Molecules. 2018 Apr 24;23(5):989. doi: 10.3390/molecules23050989 (PMC6099561; doi:10.3390/molecules23050989)
Supplement: Supplementary File 1 [file molecules-23-00989-s001.zip › figure and table PDF╬─╝■ ╡┌╢■┤╬╨▐╕─╕σ/Table 3.doc.pdf]

Table 3. Calibration curves of 5 standard chemicals

Standard chemicals	Regression equation	R^2	Linear range
Total flavonoids	y = 0.5899x + 0.0199	0.9999	0.00–2.00
Rosmarinic acid	y = 39999x + 0.0031	0.9999	0.000209–0.001045
Caffeic acid	y = 49130x – 0.0422	0.9999	0.000108–0.00054
Salviaflaside	y = 23282x – 0.044	0.9999	0.00022–0.0011
Hyperoside	y = 33699x – 0.0094	0.9999	0.00011–0.00055

y: peak area; x: concentration of chemicals (mg mL^{-1}).
